# Preparation and Characterization of AgNPs In Situ Synthesis on Polyelectrolyte Membrane Coated Sericin/Agar Film for Antimicrobial Applications

**DOI:** 10.3390/ma11071205

**Published:** 2018-07-13

**Authors:** Liying Liu, Rui Cai, Yejing Wang, Gang Tao, Lisha Ai, Peng Wang, Meirong Yang, Hua Zuo, Ping Zhao, Hong Shen, Ahmad Umar, Huawei He

**Affiliations:** 1 State Key Laboratory of Silkworm Genome Biology, Southwest University, Chongqing 400715, China; l3341345@email.swu.edu.cn (L.L.); taogang@email.swu.edu.cn (G.T.); als123@email.swu.edu.cn (L.A.); yangmeirong@email.swu.edu.cn (M.Y.); zhaop@swu.edu.cn (P.Z.); 2 College of Biotechnology, Southwest University, Chongqing 400715, China; cairui0330@email.swu.edu.cn (R.C.); modelsums@email.swu.edu.cn (P.W.); 3 College of Pharmaceutical Sciences, Southwest University, Chongqing 400715, China; zuohua@swu.edu.cn; 4 Chongqing Engineering and Technology Research Center for Novel Silk Materials, Southwest University, Chongqing 400715, China; 5 College of Resources and Environment, Southwest University, Chongqing 400715, China; shenhong@swu.edu.cn; 6 Department of Chemistry, College of Science and Arts and Promising Centre for Sensors and Electronics Devices (PCSED), Najran University, P.O. Box: 1988, Najran 11001, Saudi Arabia; umahmad@nu.edu.sa

**Keywords:** silver nanoparticle, sericin, agar, polyelectrolyte membrane, antimicrobial activity

## Abstract

Antibacterial materials are of great importance in preventing bacterial adhesion and reproduction in daily life. Silver nanoparticle (AgNP) is a broad-spectrum antibacterial nanomaterial that has attracted significant attentions for its ability to endow natural materials with antibacterial ability. Silk sericin (SS) has a great advantage for biomaterial application, as it is a natural protein with excellent hydrophilicity and biodegradability. In this study, we prepared AgNPs and polyelectrolyte membrane (PEM) modified SS/Agar films through the layer-by-layer adsorption technique and ultraviolet-assisted AgNPs synthesis method. The film was well characterized by scanning electron microscopy, energy dispersive spectroscopy, X-ray diffraction, fourier transform infrared spectroscopy, X-ray photoelectron spectroscopy. Other properties such as water contact angle, wettability and tensile strength, the release of silver were also studied. The antimicrobial activity of AgNPs-PEM-SS/Agar film was investigated against *Escherichia coli* and *Staphylococcus* aureus as the model microorganisms by the inhibition zone and bacterial growth curve assays. The results suggested that the AgNPs-PEM-SS/Agar film had excellent mechanical performance, high hydrophilicity, prominent water absorption ability, as well as outstanding and durable antibacterial activity. Therefore, the prepared novel AgNPs-PEM-SS/Agar composite film is proposed as a potentially favorable antibacterial biomaterial for biomedical applications.

## 1. Introduction

Over the years, the increasing bacterial infections have caused serious health issues, owing to the enhanced penetration and antibiotic resistance of bacteria [1]. Nowadays, nanostructured materials are considered as promising, novel antibacterial agents for biomedical applications because of their high surface-to-volume ratio and unique physical and chemical capability on the nanoscale level [2]. Silver nanoparticle (AgNP), a well-known nanomaterial with broad antibacterial activity [3,4], can endow natural materials with the antimicrobial activity [5]. Ultraviolet (UV)-assisted synthesis technique is developed to immobilize AgNPs in situ on the surface of materials [6]. It is one of the most green and facile approaches to prepare AgNPS without expensive instruments [7] and toxic chemicals [8]. 

Silk sericin (SS), a natural macromolecular protein, constitutes silk protein together with silk fibroin [9]. Sericin consists of 18 amino acids, most of which have polar groups like carboxyl, amino, and hydroxyl groups [10]. It has high contents of serine (40%) and glycine (16%) [11,12]. Serine is known as the principle moisturizing factor of human skin [13,14]. Therefore, sericin has good hydrophilicity and skin moisturizing effect [15,16,17]. In addition, sericin has anti-oxidant, anti-coagulant activity [18] and can enhance the attachment and proliferation of mammalian cells such as human fibroblasts [19]. Sericin has attracted a huge deal of attentions for its excellent properties in cosmetics [20] and biomedical application [21]. However, sericin is naturally fragile which limits its applications [22]. To improve the mechanical property, sericin is often co-polymerized, cross-linked or blended with other substances [23]. Chemical agents like glutaraldehyde used for chemical crosslinking could be potentially toxic [24]. The blending of sericin with polymers, such as gelatin, polyvinyl alcohol has been proved to effectively enhance the mechanical strength of sericin [25]. Agar is a highly hydrophilic polysaccharide with high mechanical strength, which has promising application in biomaterials such as cell culture and wound dressing [26]. In this study, Agar was blended with sericin to improve its mechanical performance. On this basis, AgNPs were synthesized *in situ* on the SS/Agar film to impart antibacterial ability for the prepared material which expand the potential of SS/Agar film in food packaging, biomedical field or tissue engineering [27]. However, since the Ag^+^ binding sites on the surface of SS/Agar film are very limited, only few AgNPs can be synthesized, resulting in low antibacterial activity. Moreover, the oxidization or falling off of AgNPs synthesized on the material’s surface may further lower its antibacterial effects. Layer-by-layer (LbL) assembly has emerged as the most versatile and popular approach to fabricate polyelectrolyte membrane (PEM) which could provide a three-dimensional structure on the material’s surface to promote the generation of AgNPs and protect the synthesized AgNPs from oxidization or falling off [28]. In this technique, two oppositely charged polyelectrolytes are alternately deposited on a support surface via van der Waals forces and electrostatic attractions [29]. Polyacrylic acid (PAA) and poly (dimethyldiallylammonium chloride) (PDDA) are two common polymers used to form PEM on the surface of materials [30,31]. PEM could not only provide more silver ion binding sites for the synthesis of high-density AgNPs, but also prevent AgNPs from oxidization or falling off to make the synthesized AgNPs more stable.

In this work, we prepared a PAA/PDDA/PAA structure on the surface of SS/Agar film via the LbL assembly technique, and then synthesized AgNPs in situ in the PEM via UV irradiation. The prepared AgNPs-PEM-SS/Agar films were characterized by scanning electron microscopy (SEM), energy dispersive spectroscopy (EDS), X-ray diffractometry (XRD), fourier transform infrared spectroscopy (FT-IR) and X-ray photoelectron spectroscopy (XPS). The water contact angle and mechanical property were also studied. The antimicrobial property of AgNPs-PEM-SS/Agar films were investigated against the model microorganisms, Escherichia coli (E. coli) and Staphylococcus aureus (S. aureus) by the inhibition zone and bacterial growth curve assays. Inductively coupled plasma atomic absorption spectroscopy (ICPAAS) was used to measure the release of silver from the AgNPs-PEM-SS/Agar film. The prepared films have shown great potentials in antimicrobial applications such as wound dressing, artificial skin and tissue engineering.

## 2. Materials and Methods

### 2.1. Materials

Bombyx mori cocoons were provided by the State Key Laboratory of Silkworm Genome Biology, Southwest University, China. Agar, Poly (ethyleneimine) (PEI, MW 750,000 Da), PAA (MW 3000 Da), PDDA (MW 200,000–350,000 Da) and silver nitrate (AR, 99.99%) were bought from Aladdin (Shanghai, China). All other chemicals in this study were of analytical grade and used directly without further purification.

### 2.2. Fabrication of AgNPs Modified PEM-SS/Agar Film

**Figure 1:**
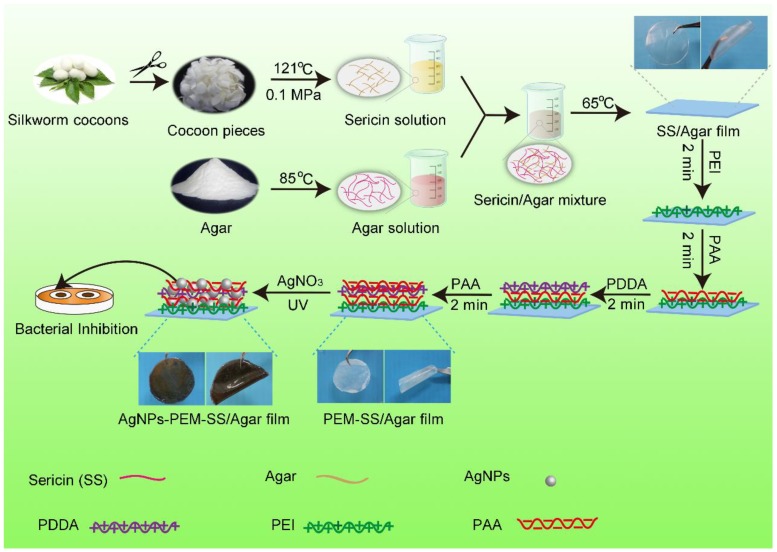
Preparation and antibacterial analysis of AgNPs-PEM-SS/Agar film.

The fabrication and antibacterial analysis of the AgNPs-PEM-SS/Agar composite film were shown in Figure 1. Sericin was extracted according to a previous report [32]. Agar was dissolved to a final concentration of 2% (w/t) with agitation at 85 °C. Sericin solution (2%, w/v) and agar solution (2%, w/v) were well mixed in a volume ratio of 1:1 at 65 °C. Then the mixture were cast in a clean petri dish and dried at 65 °C to form SS/Agar blend film. Next, the blend film was immersed into PEI solution (2%, w/v) for 2 min to functionalize the surface with positive charges. After washing with water, the film was treated alternately with the negative charged PAA (2%, w/v) and the positively charged PDDA (2%, w/v) to form one layer of PEM on the film’s surface. Finally, the PEM-SS/Agar film was immersed in 20 mM silver nitrate and then irradiated with 365 nm UV light for different times to synthesize AgNPs in situ on the surface of the composite film. The prepared AgNPs-PEM-SS/Agar films were dried at room temperature for further analysis.

### 2.3. Material Characterizations

The morphology of SS/Agar, PEM-SS/Agar and AgNPs-PEM-SS/Agar films were imaged on a JEOL JSM-6510LV SEM (Tokyo, Japan). At the same time, EDS spectra were collected on an INCA X-Max 250 to analyze the chemical elements of the films. Image J was used to analyze the size distribution of the synthesized AgNPs. The XRD patterns of SS/Agar, PEM-SS/Agar and AgNPs-PEM-SS/Agar films were recorded on an X’Pert powder X-ray diffractometer (PANalytical, Almelo, Netherland) with a 2θ range of 10–80°. The FT-IR spectra of SS/Agar, PEM-SS/Agar and AgNPs-PEM-SS/Agar films were analyzed from 4000 to 800 cm^−1^ on a Nicolet iz10 FT-IR spectrometer (Waltham, MA, USA) at a resolution of 2 cm^−1^. 

### 2.4. XPS Analysis

XPS measurement was performed on a Thermo Fisher ESCALAB 250 (Waltham, MA, USA) with an Al Kα X-ray source to determine the chemical state of AgNPs and the surface element composition.

### 2.5. Water Contact Angle

The surface hydrophobicity of the composite film was determined by a Krüss DSA100 water contact angle analyzer (Hamburg, Germany). The samples were mounted on a horizontal mobile station. About 4 μL water was dropped on the film and the contact angles on both sides of the water droplets were measured. Five measurements were averaged for each sample.

### 2.6. Swelling Ratio

The initial dry films (3 cm × 3 cm) were weighted (m_1_) and then immersed into water or PBS buffer (pH 7.4). Afterwards, the swollen samples were removed from solution at different intervals and weighted after the removal of the extra solution on the surface of the film. The weight at this time was denoted as m_2_. Three replicates were made for each sample. The swelling ratio was calculated as follows:

Swelling ratio (%) = (m_2_ − m_1_)/m_1_ × 100% (1)

### 2.7. Mechanical Property

Mechanical properties were studied on an AG-X plus (SHIMADZU, Kyoto, Japan) equipped with a 1000-N load cell. The films were sliced into 4 cm × 1 cm (length × width) and pulled at a crosshead speed of 3 mm/min and elongated until breaking. At least eight samples were examined for each film. The ultimate tensile stress and ultimate elongation of films were obtained according to the fracture data.

### 2.8. Inhibition Zone Assay 

The typical Gram-positive bacteria and Gram-negative bacteria, E. coli and S. aureus, were used in this experiment to investigate the antibacterial activity of the prepared films with the inhibition zone assay [33]. The bacteria were cultured in Luria-bertani (LB) medium (pH 7.4) at 37 °C with 220 rpm shaking until the optical density (OD) at 600 nm (OD_600_) of 1.0. Then, the bacteria (200 μL) were spread on a solid agar medium in the presence of circular SS/Agar, PEM-SS/Agar and AgNPs-PEM-SS/Agar films (d = 11 mm), and then incubated at 37 °C overnight. The diameters of the formed inhibition zones were measured to compare the antibacterial activities of the films. Three replicates of each measurement were made to ensure the accuracy of the experiments.

### 2.9. Growth Curve Assay 

The bacteria were inoculated into 10 mL of LB medium and cultured with 220 rpm shaking at 37 °C in the presence of circular SS/Agar, PEM-SS/Agar and AgNPs-PEM-SS/Agar films (3 cm × 3 cm). At different time intervals, 0.5 mL bacterial suspensions were collected to measure OD_600_. Then, the bacterial growth curve was determined as the recorded OD_600_ at different time points. All the measurements were made in three repetitions.

### 2.10. Antibacterial Stability 

For the antibacterial stability test, AgNPs-PEM-SS/Agar films (1 cm × 1 cm) were soaked into PBS buffers (pH 4, 7.4 or 9) for 24 h. Then, the films were removed from PBS buffers and applied for the bacterial growth assay as described above.

### 2.11. Degradation of AgNPs-PEM-SS/Agar Film

The degradation of AgNPs-PEM-SS/Agar film was determined as the mass loss percentage of the film after immersion into PBS buffers (pH 4, 7.4, 10) at 37 °C. First, AgNPs-PEM-SS/Agar films were soaked into PBS buffers for 24 h, and then dried at 60 °C for 24 h. The dried films were weighted as W_1_. Then, the films were immersed into PBS buffers at 37 °C, removed at given time points and dried at 65 °C for 24 h to obtain the residual mass (W_2_). The mass loss rate was calculated as follows:

Mass loss rate (%) = (W1 − W2)/W1 × 100%(2)

### 2.12. Release of Silver

The release of silver from the AgNPs-PEM-SS/Agar film was measured on a Z-5000 atomic absorption spectrometer (Hitachi, Japan) using the N_2_O acetylene flame AAS method. The AgNPs-PEM-SS/Agar films with a dimension of 1.5 cm in length and 1.5 cm in width were immersed into 0.01M PBS buffer (pH 7.4) at 37 °C. The PBS solution was collected for ICPAAS measurement at regular intervals. Then fresh PBS buffer was added after each measurement. Different AgNO_3_ concentrations (0.5–10 mg/L) were prepared for the calibration curve. All experiments were carried out in triplicate.

### 2.13. Statistics

All experiments were carried out at least in triplicate, and the data were presented as the mean ± standard deviation (SD).

## 3. Results and Discussion

### 3.1. SEM, EDS and XRD

**Figure 2:**
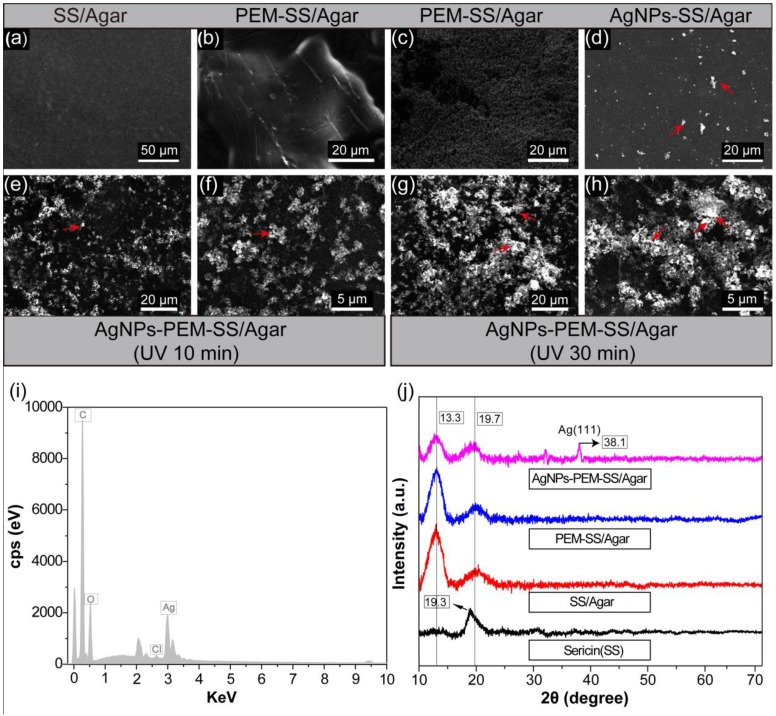
Surface morphologies of SS/Agar (**a**); PEM-SS/Agar (**b**); cross-section of PEM-SS/Agar films (**c**); AgNPs-SS/Agar film (10 min irradiation) (**d**); AgNPs-PEM-SS/Agar film (10 min (**e**,**f**) and 30 min (**g**,**h**) irradiation), (**f**,**h**) are the high magnification of (**e**,**g**), respectively; EDS spectrum of AgNPs-PEM-SS/Agar film (**i**); XRD patterns of different films (**j**).

The surface morphologies of different films were investigated by SEM (Figure 2). SS/Agar film had a smooth surface, indicating that sericin and agar were well blended. Figure 2b,c showed the SEM images of PEM-SS/Agar film, clearly indicating the formation of PEM on the surface of SS/Agar film. Figure 2d,e showed the morphologies of AgNPs-SS/Agar and AgNPs-PEM-SS/Agar films, respectively. It was noted that under the same condition, PEM-SS/Agar film had a higher density of AgNPs than that of SS/Agar film, indicating that PEM can facilitate the synthesis and growth of AgNPs. This is because PEM can provide a 3-D space for the adsorption and binding of more silver ions, thus promote the reduction of Ag^+^ to Ag^0^ under the irradiation of UV [34]. As shown in Figure 2e,g, as the UV irradiation time increased from 10 minutes (Figure 2e) to 30 minutes (Figure 2g), more and more AgNPs occurred on the AgNPs-PEM-SS/Agar film, suggesting irradiation time could regulate the synthesis of AgNPs. The particle size analysis showed the size of AgNPs formed on the composite film was almost below 100 nm. Most of AgNPs had a size range of 50–80 nm (Figure S1). The result suggested AgNPs were successfully synthesized on the composite film.

Figure 2i shows the EDS spectra of AgNPs-PEM-SS/Agar film. The result showed an apparent peak of silver element, which further indicated the generation of AgNPs. Figure 2j shows the XRD patterns of sericin, SS/Agar, PEM-SS/Agar and AgNPs-PEM-SS/Agar films, respectively. A characteristic peak at about 19.2° was assigned to the diffraction of sericin [35]. The other three films had two characteristic peaks at about 13.3° and 19.7°, which were attributed to agar and sericin, respectively. After PEM coating and AgNPs modification, the XRD patterns of the film did not change obviously, indicating that these modifications did not affect the crystal structure of SS/Agar film. For AgNPs-PEM-SS/Agar film, the peak at 38.1° was assigned to the (111) planes of crystalline Ag, indicating that the synthesized AgNPs had good crystal structure. The high crystallinity, high density and good dispersivity of AgNPs can effectively improve the antibacterial activity of SS/Agar film. 

### 3.2. FT-IR 

**Figure 3:**
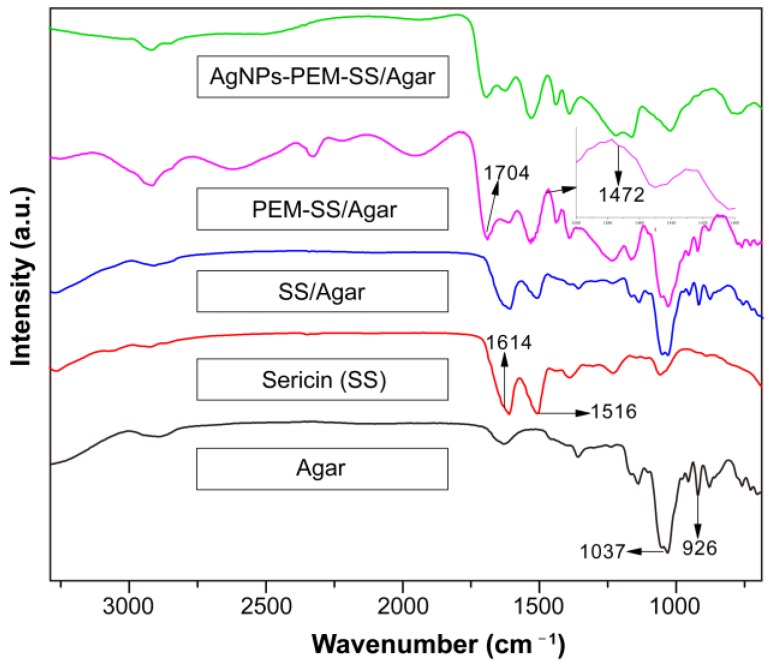
FT-IR spectra of agar, sericin, SS/Agar film, PEM-SS/Agar, and AgNPs-PEM-SS/Agar films.

FT-IR was carried out to characterize the structure of the films. As shown in Figure 3, two peaks at 1516 cm^−1^ and 1614 cm^−1^ appeared in all samples except pure Agar film, which represent amid I (C=O stretching) and amid II (N–H deformation) peaks of sericin, respectively [23]. Furthermore, two peaks were observed at 1037 and 926 cm^−1^ in pure Agar film, which are the characteristic peaks of agar [36]. SS/Agar film had the characteristic peaks of sericin and agar, indicating that sericin and agar were compatible and the blending did not affect their properties. After PEM modification, two more peaks appeared at 1704 cm^−1^ and 1472 cm^−1^, which correspond to the carbonyl absorption band of PAA [37] and the methyl stretching vibration of PDDA [38], respectively. This result further confirmed the presence of PEM. In addition, the amide peaks of sericin in SS/Agar films had no significant change after PEM coating and AgNPs modification, indicating that these modifications would not affect the structure of SS/Agar film. 

### 3.3. XPS

**Figure 4:**
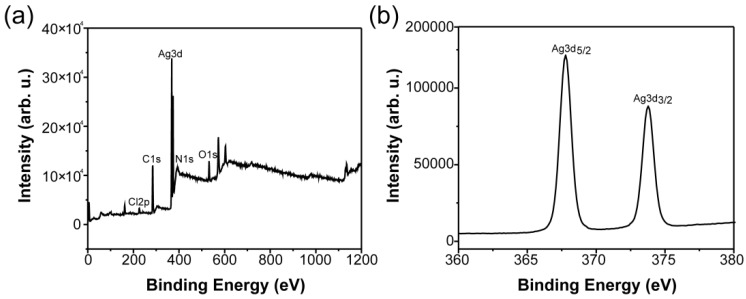
XPS spectra of AgNPs-PEM-SS/Agar film (**a**) and Ag (3d) electron binding energy spectrum (**b**).

Figure 4a showed the XPS spectra of the AgNPs-PEM-SS/Agar film. In addition to C (1s), N (1s), O (1s), and Cl (2p), the XPS spectrum of the composite film also contained element Ag (3d), which further confirmed the successful synthesis of AgNPs on the surface of the film. Figure 4b was the high definition XPS spectra of Ag 3d electron binging energy. Two peaks appeared at 368 eV and 374 eV corresponding to Ag 3d5/2 and Ag 3d3/2 binding energy, respectively [39,40]. Compared with the binding energy of Ag^0^ (368.3 eV and 374.3 eV), the peaks of Ag (3d) red-shifted, which may be attributed to the formation of AgNPs [41]. 

### 3.4. Wettability and Water Uptake Ability

**Figure 5:**
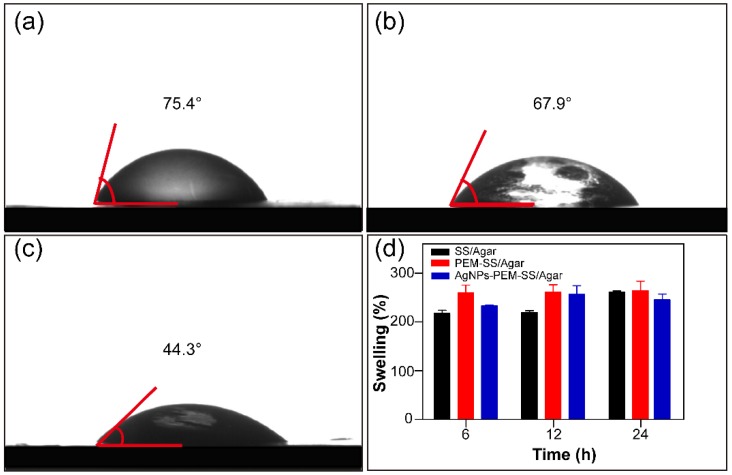
Water contact angles of SS/Agar (**a**), PEM-SS/Agar (**b**), AgNPs-PEM-SS/Agar films (**c**) and swelling ratio of different films at different times (**d**).

Water contact angle was used to characterize the wettability of the films. The water contact angle of SS/Agar film was 75.4° (Figure 5a), indicating it was hydrophilic. The water contact angle of PEM-SS/Agar was 67.9° (Figure 5b), indicating PEM coating increased the hydrophilicity of SS/Agar film. After AgNPs modification, the water contact angle of PEM-SS/Agar film decreased to 44.3° (Figure 5c), suggesting that AgNPs greatly increased the surface hydrophilicity of PEM-SS/Agar film. This may be AgNPs modification improved the surface coarseness of the film which is conducive to the increase of hydrophilicity [42]. In addition, AgNPs are partially oxidized to Ag^+^ in the aqueous solution, and Ag^+^ could form hydrated silver ions, which result in the increase of hydrophilicity [43,44,45].

To further illustrate the hydrophilicity of the films, the swelling of the films were tested, as shown in Figure 5d. The swelling ratios of the films at different times were more than 200%, indicating that the films had good hydrophilicity and durable moisturizing performance. These features are essential for biological applications. In addition, we tested the swelling ratio of the films in PBS buffer (pH 7.4). The result showed that the swelling ratio of SS/Agar and PEM-SS/Agar composite film in PBS buffer were similar to those in water. The swelling ratio of both composite films were more than 200%. For AgNPs-PEM-SS/Agar film, the swelling ratio in PBS buffer was slightly lower than that in water (Figure S2). This was probably because the release of silver in PBS buffer was faster than that in water. Our result indicated PBS buffer had little effect on the release of silver. 

### 3.5. Mechanical Property

**Figure 6:**
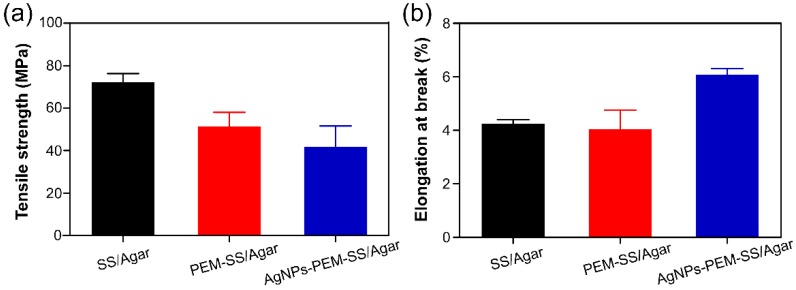
Mechanical properties of the films: (**a**) tensile strength and (**b**) elongation at break.

The mechanical properties of SS/Agar, PEM-SS/Agar and AgNPs-PEM-SS/Agar films were investigated by the tensile strength (N/mm^2^) and elongation at break (%), as shown in Figure 6. The tensile strength of SS/Agar, PEM-SS/Agar and AgNPs-PEM-SS/Agar films were 72.08 MPa, 51.09 MPa, and 41.74 MPa, and the elongation at break were 4.23%, 4.05% and 6.08%, respectively. The results suggested that AgNPs modification enhanced the strain of SS/Agar film but reduced its strength. The elongation at break represents the flexibility of a film [46]. The increase in the flexibility of the prepared film is suitable for active packaging, biomedical field or tissue engineering application.

### 3.6. Inhibition Zone Assay

**Figure 7:**
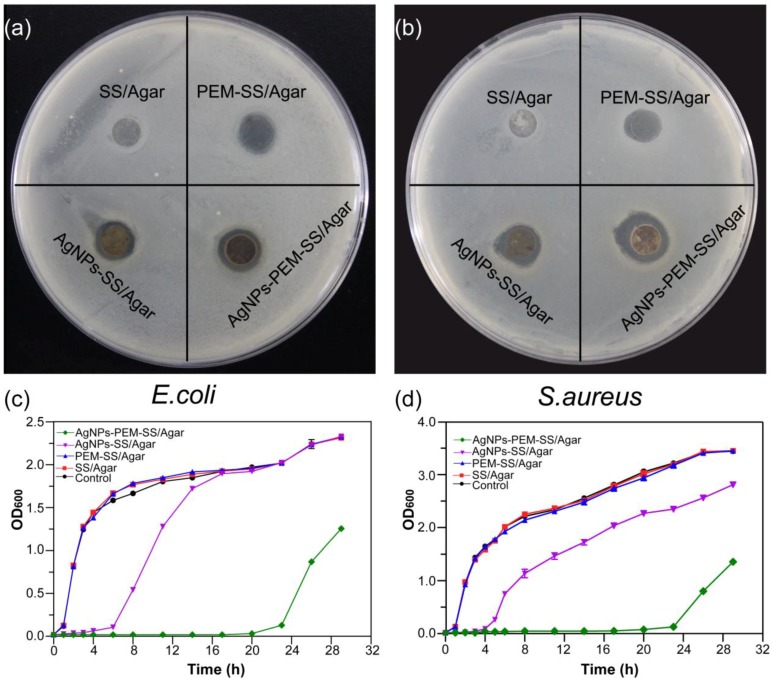
The inhibition zones of SS/Agar, PEM-SS/Agar, AgNPs-SS/Agar, AgNPs-PEM-SS/Agar films against E. coli (**a**) and S. aureus (**b**); Growth curves of *E. coli* (**c**) and *S. aureus* (**d**).

Figure 7a,b showed the inhibition zones of different films against *E. coli* and *S. aureus*, respectively. No inhibition zone appeared for the SS/Agar film, indicating that SS/Agar film cannot inhibit bacterial growth. AgNPs-SS/Agar and AgNPs-PEM-SS/Agar films formed clear inhibition zone, indicating the films had good bactericidal effect. It was noted that the bactericidal effect of AgNPs-PEM-SS/Agar film was better than that of AgNPs-SS/Agar film, indicating PEM coating increased synthesized AgNPs and thus enhanced the antibacterial ability of AgNPs-SS/Agar film. In addition, the PEM-SS/Agar film also had an inconspicuous inhibition zone. It was probably due to the leakage of cationic PAA which contains a large number of acidic groups and could inhibit bacterial growth in humid environment [47]. The diameters of the inhibition zone of different films were shown in Table 1.

### 3.7. Bacterial Growth Curve

Bacterial growth curves were used to further investigate the antibacterial activity of the films. Figure 7c,d showed the growth curves of *E. coli* and *S. aureus* in the presence of different films. Compared with the control, SS/Agar and PEM-SS/Agar films almost did not affect the bacterial growth. AgNPs-SS/Agar and AgNPs-PEM-SS/Agar films exhibited good antibacterial activity as the films significantly retarded the bacterial growth. It was noted that the inhibitory effect of AgNPs-PEM-SS/Agar film was more effective than that of AgNPs-SS/ Agar film, which was consistent with the observation of the inhibition zone assay. This result further confirmed that PEM coating enhanced the antibacterial activity of AgNPs-SS/Agar films. 

### 3.8. Antibacterial Stability

**Figure 8:**
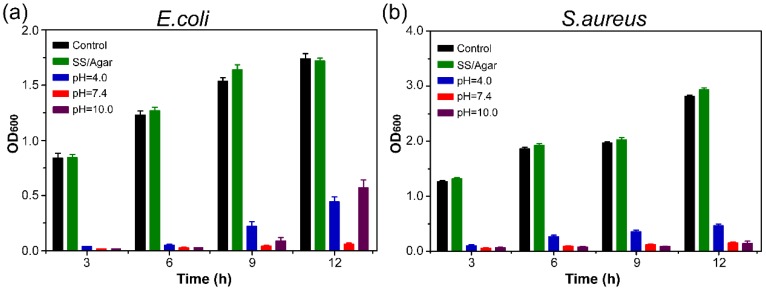
Antibacterial stability of AgNPs-PEM-SS/Agar film against *E. coli* (**a**) and *S. aureus* (**b**).

The antibacterial stability of AgNPs-PEM-SS/Agar film was determined, as shown in Figure 8. Compared with the control, AgNPs-PEM-SS/Agar film obviously inhibited the bacterial growth both for E. coli (Figure 8a) and S. aureus (Figure 8b) after treatment under different pH conditions for 12 h, indicating the treatment did not affect the antibacterial ability of the film. This result suggested that the prepared film had stable antibacterial ability, which is advantageous for its application in different pH conditions.

### 3.9. Degradation of AgNPs-PEM-SS/Agar Film

**Figure 9:**
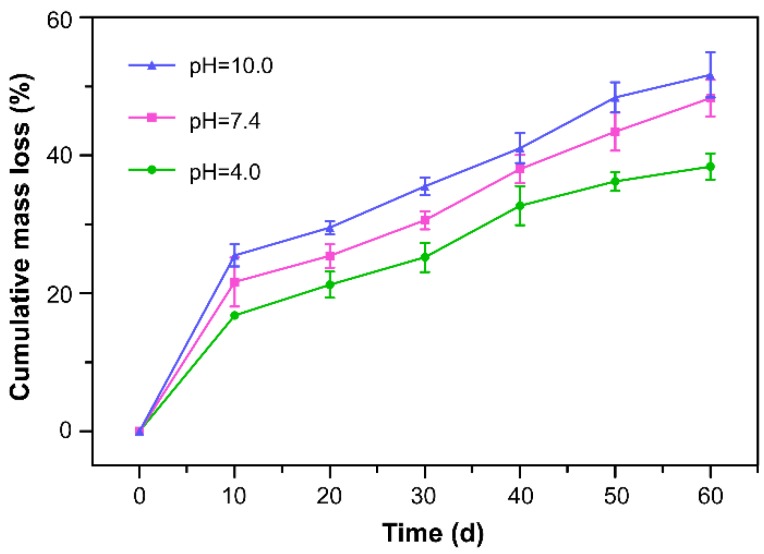
Degradation of AgNPs-PEM-SS/Agar film at pH 4.0, 7.4, 10.0.

Biodegradability is a desirable property of environmentally friendly biomaterials. Also, the controllable degradation of antibacterial film is favorable for the release of silver from the film to achieve long-term inhibiting bacterial effects. Figure 9 showed the degradation of AgNPs-PEM-SS/Agar films at different pH (4.0, 7.4, 10.0). After 60 days of treatment, the mass loss of the film was about 37% at pH 4.0, about 45% at pH 7.4, and about 55% at pH 10.0, respectively. This result suggested that the film was biodegradable. The mass loss of the film in pH 10.0 was faster than that in pH 4.0. It is likely sericin has an isoelectric point of 3.8 and high contents of acidic amino acid residues. 

### 3.10. Release of Silver

**Figure 10:**
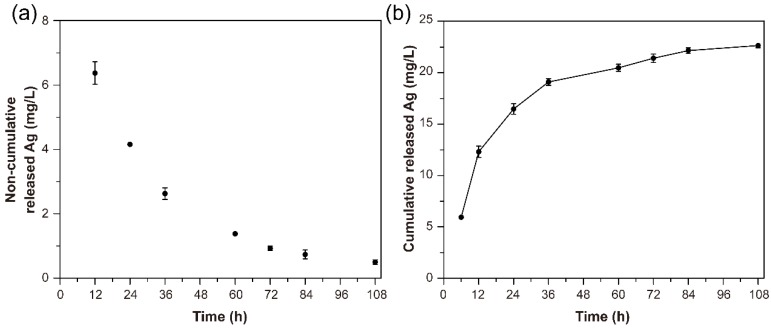
Release profile of silver from the AgNPs-PEM-SS/Agar film in PBS buffer. (**a**) non-cumulative and (**b**) cumulative release of Ag after treatment at 37 °C for 108 h.

The AgNPs-PEM-SS/Agar film exerts antibacterial effect by releasing the bound Ag^+^/AgNPs into environment. To evaluate the release of Ag^+^/AgNPs, ICPAAS were performed. Figure 10a,b indicated the Ag release profiles of AgNPs-PEM-SS/Agar film in PBS buffer. The result showed the release rate of silver decreased as time went on. Within the first 12 h, the Ag release rate was faster than that after 12 h, which may be due to the fact that the Ag at the edge of the sample released easily into PBS buffer in a short time. The release of silver lasted up to 108 h, indicating the durable antibacterial activity of AgNPs-PEM-SS/Agar film. Compared with other antibacterial materials, the AgNPs-PEM-SS/Agar films showed a more durable antibacterial ability due to the presence of PEM [48,49,50,51]. In a previous work, we have prepared AgNPs modified sericin/glycerol blend film (AgNPs/SS/Gly) without PEM and tested the Ag release [52]. Although there are some differences in the composition of the two composite films, the composition of the films (glycerol/agar) almost does not affect AgNPs synthesis and Ag release. Therefore, we compared Ag release of AgNPs/SS/Gly film with AgNPs-PEM-SS/Agar film prepared in this work. The result showed after 24 h, the release rate of Ag in the film without PEM was higher than that of the film with PEM, indicating the three-dimensional structure created by PEM could reduce Ag release. The prepared AgNPs and PEM modified SS/Agar material has shown efficient antibacterial capabilities and great potentials in biomedical application. 

## 4. Conclusions

In this work, we prepare the AgNPs-PEM-SS/Agar composite film through a simple and efficient layer-by-layer assembly technique and UV-assisted AgNPs synthesis method. PEM provide a three-dimensional space to promote the formation of high-density AgNPs as well as protect AgNPs from oxidation and falling-off, thus efficiently improve the antibacterial activity of the film. The AgNPs-PEM-SS/Agar film has good mechanical properties, high hydrophilicity, excellent water absorption ability, as well as excellent and durable antibacterial capability. Therefore, the novel material has a very promising prospect in antibacterial applications such as tissue engineering and wound healing dressing for burns and surgical wounds.

## Figures and Tables

**Table 1:** Diameters of the inhibition zones of SS/Agar, PEM-SS/Agar, AgNPs-SS/Agar and AgNPs-PEM-SS/Agar films against *E. coli* (a) and *S. aureus* (b).

Bacteria	Control (cm)	PEM-SS/Agar (cm)	AgNPs-SS/Agar (cm)	AgNPs-PEM-SS/Agar (cm)
*E. coli*	1.10 ± 0.00	1.31 ± 0.01	1.51 ± 0.03	1.68 ± 0.04
*S. aureus*	1.10 ± 0.00	1.20 ± 0.10	1.65 ± 0.05	1.98 ± 0.03

## Supplementary Materials

The following are available online at http://www.mdpi.com/1996-1944/11/7/1205/s1, Figure S1: SEM image of AgNPs-PEM-SS/Agar film (A) and size distribution analysis of AgNPs (B), Figure S2. Swelling ratio of different films in PBS buffer (pH 7.4) at different time.
