# Supplementary material for: Preparation and Characterization of AgNPs In Situ Synthesis on Polyelectrolyte Membrane Coated Sericin/Agar Film for Antimicrobial Applications
Source: Materials (Basel). 2018 Jul 13;11(7):1205. doi: 10.3390/ma11071205 (PMC6073696; doi:10.3390/ma11071205)
Supplement: Supplementary file 1 [file materials-11-01205-s001.pdf]

Preparation and Characterization of AgNPs in Situ Synthesis on Polyelectrolyte Membrane Coated Sericin/Agar film for Antimicrobial Applications

Liying Liu [1], Rui Cai [2], Yejing Wang [1,2,*], Gang Tao [1], Lisha Ai [1], Peng Wang [2], Meirong Yang [1], Hua Zuo [3], Ping Zhao [1,4], Hong Shen [5], Ahmad Umar [6], and Huawei He [1,4,*]

1. State Key Laboratory of Silkworm Genome Biology, Southwest University, Chongqing 400715, China; l33341345@email.swu.edu.cn (L.L.); taogang@email.swu.edu.cn (G.T.); als123@email.swu. edu.cn (L.A.); yangmeirong@email.swu.edu.cn (M.Y.); zhaop@swu.edu.cn (P.Z.)
2. College of Biotechnology, Southwest University, Chongqing 400715, China; cairui0330@email.swu.edu.cn (R.C.); modelsums@email.swu.edu.cn (P.W.)
3. College of Pharmaceutical Sciences, Southwest University, Chongqing 400715, China; zuohua@swu.edu.cn
4. Chongqing Engineering and Technology Research Center for Novel Silk Materials, Southwest University, Chongqing 400715, China
5. College of Resources and Environment, Southwest University, Chongqing 400715, China; shenhong@swu.edu.cn
6. Department of Chemistry, College of Science and Arts and Promising Centre for Sensors and Electronics Devices (PCSED), Najran University, P.O. Box: 1988, Najran 11001, Saudi Arabia; umahmad@nu.edu.sa

* Correspondence: yjwang@swu.edu.cn (Y.W.); hehuawei@swu.edu.cn (H.H.); Tel.: +86-23-6825-1575 (H.H.)

Received: 5 June 2018; Accepted: 9 July 2018; Published: date

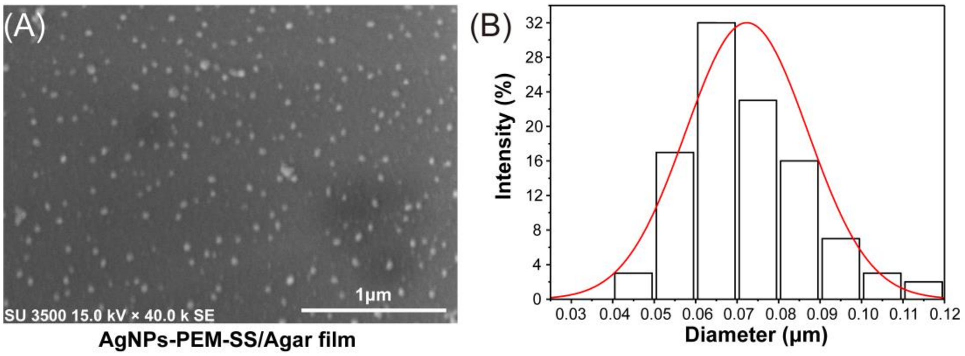

Figure S1. Scanning electron microscopy (SEM) image of AgNPs-PEM-SS/Agar film (**A**) and size distribution analysis of AgNPs (**B**).

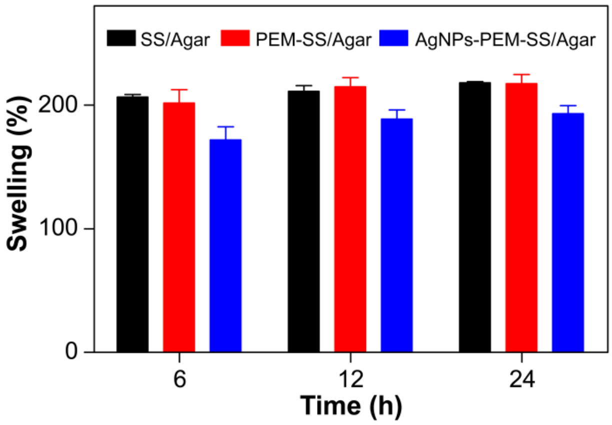

Figure S2. Swelling ratio of different films in PBS buffer (pH 7.4) at different time.
